# Self-care tooling innovation in a disabled kea (*Nestor notabilis*)

**DOI:** 10.1038/s41598-021-97086-w

**Published:** 2021-09-10

**Authors:** Amalia P. M. Bastos, Kata Horváth, Jonathan L. Webb, Patrick M. Wood, Alex H. Taylor

**Affiliations:** 1 School of Psychology, The University of Auckland, Private Bag 92019, Auckland, 1142 New Zealand; 2 Doctoral School of Psychology, ELTE Eötvös Loránd University, Izabella Utca 46, 1064 Budapest, Hungary; 3 Institute of Psychology, ELTE Eötvös Loránd University, Izabella Utca 46, 1064 Budapest, Hungary; 4 Brain, Memory and Language Research Group, Institute of Cognitive Neuroscience and Psychology, Research Centre for Natural Sciences, Magyar tudósok körútja 2, 1117 Budapest, Hungary

**Keywords:** Psychology, Zoology

## Abstract

Tooling is associated with complex cognitive abilities, occurring most regularly in large-brained mammals and birds. Among birds, self-care tooling is seemingly rare in the wild, despite several anecdotal reports of this behaviour in captive parrots. Here, we show that Bruce, a disabled parrot lacking his top mandible, deliberately uses pebbles to preen himself. Evidence for this behaviour comes from five lines of evidence: (i) in over 90% of instances where Bruce picked up a pebble, he then used it to preen; (ii) in 95% of instances where Bruce dropped a pebble, he retrieved this pebble, or replaced it, in order to resume preening; (iii) Bruce selected pebbles of a specific size for preening rather than randomly sampling available pebbles in his environment; (iv) no other kea in his environment used pebbles for preening; and (v) when other individuals did interact with stones, they used stones of different sizes to those Bruce preened with. Our study provides novel and empirical evidence for deliberate self-care tooling in a bird species where tooling is not a species-specific behaviour. It also supports claims that tooling can be innovated based on ecological necessity by species with sufficiently domain-general cognition.

## Introduction

Tooling—deliberately generating a mechanical interface by using an object to manipulate another target or surface^1,2^—is a complex embodied form of tool use which has been documented in only a few species^1,3,4^. When tooling, an animal and the tooling object are transformed into a single body-plus-object system^1,2^, performing contextually appropriate, embodied problem-solving^1,2,5^. Flexible tool use, including tooling, often emerges in the form of behavioural innovations, where animals develop a novel behaviour in response to situational changes^6^. Tool use behavior—including but not limited to tooling—has been regarded as a marker of complex cognition across birds and mammals^4,7,8^, a link which has led to considerable interest in the field^3,9–12^. Within birds, flexible tooling has been found most commonly in clades with large relative brain sizes, such as corvids^3,9–11,13–18^ and parrots^3,10,19–23^.

Most reports of tooling in birds revolve around foraging^9–12,14,15,20,21,23–25^. Among parrots this is more common in captive settings, for example, greater vasa parrots use small stones to scrape or break up shells, which they then ingest^23^, hyacinth macaws use wedges to manipulate nuts^20^, and Goffin’s cockatoos innovate and manufacture stick tools to retrieve out-of-reach food^19^. Despite not habitually using tools in the wild, kea parrots (*Nestor notabilis*) learn to insert sticks and other objects into traps designed for pest species such as stoats^21^, which allows them to safely access egg bait placed inside. Kea also probe at and set off unbaited traps, an apparently non-functional behaviour which may be playful or exploratory in nature^21^. In captivity, kea also readily learn to use stick tools for extractive foraging in experimental settings^26,27^.

In the wild, tooling in a self-care context appears to be rarer than in foraging contexts^28,29^. However, there are anecdotal and video reports of several parrot species innovating self-care tooling in captivity, primarily by holding sticks or other objects with their feet to scratch themselves^9,10,30^. These behaviours have not been rigorously reported in the literature and so it is unclear how they were innovated, how frequently they occur, or if reduced interactions with conspecifics in captivity reduces allopreening and so drives the individual innovation of self-care tooling.

Recently, a study reported self-care tool use in Atlantic puffins (*Fratercula arctica*), which were observed holding sticks to their bodies, possibly in order to scratch themselves^28^. This claim rests on two observations across four years of two puffins living in colonies over 7000 km apart (in Wales and Iceland), for which only one tool use instance was recorded on video. This claim has garnered significant attention from the scientific community, which has been skeptical that the study provides sufficient evidence of tool use^31–35^. Not only is the single recorded incident short in duration, lasting approximately one second, but the touching of the stick to the puffin’s chest may have been an accidental combination of two other behaviours, namely holding a stick and attempting to scratch itself^31,34^. Critics of this study^31–35^ have argued for a hypothesis-testing approach to anecdotal reports such as these, suggesting that convincing evidence for tool use in puffins should include: (i) comparisons between the number of instances where sticks are picked up and used for scratching, and instances where they are picked up but not used for scratching; (ii) evidence of subjects’ intention to scratch with a stick, for example by showing that they exhibit preferences for sticks with favorable characteristics, value previously useful tools, or perform the same behaviour repeatedly over multiple days; and (iii) comparisons between tool-using and non-tool-using individuals, where the latter should be more likely to pick up sticks without performing scratching actions than the former.

These criticisms are pertinent to our observations of apparent innovation of self-care tooling by a disabled kea parrot, a species where tooling is not a species-specific behaviour. Bruce, a wild-born male with an estimated age of 8 years, is disabled due to him missing the upper part of his bill. This means that he struggles to perform basic functions kea use their powerful beaks for, such as eating and preening. Bruce appears to use small pebbles (which he typically takes from a gravel path in the aviary, using his lower mandible to scoop them up) to preen himself. Pebbles are wedged between his lower mandible and tongue, and moved along his feathers. This appears analogous to other subjects’ clasping and grinding of feathers between their upper and lower mandibles.

Here, we aim to provide evidence for Bruce’s deliberate self-care tooling behaviour from repeated observations, by creating a scientifically rigorous way to report self-care tooling. In line with critiques of the puffin report, we hypothesize that this behaviour could be considered deliberate rather than incidental if our data indicate that: (i) Bruce’s instances of pebble manipulations occur simultaneously with preening more often than not; (ii) he performs this preening tooling behaviour repeatedly and specifically with pebbles, retrieving or replacing lost preening pebbles; (iii) he uses pebbles with particular characteristics which can afford this preening function; (iv) he preens with a pebble more often than other individuals with typical beak morphology housed in the same aviary, who should be less likely than him to combine object manipulation and preening behaviours into a single action; and (v) the types of objects he interacts with are different to those selected by other individuals, who do not use pebbles as preening tools.

## Results

### Do Bruce’s pebble manipulations co-occur with preening?

**Figure 1:**
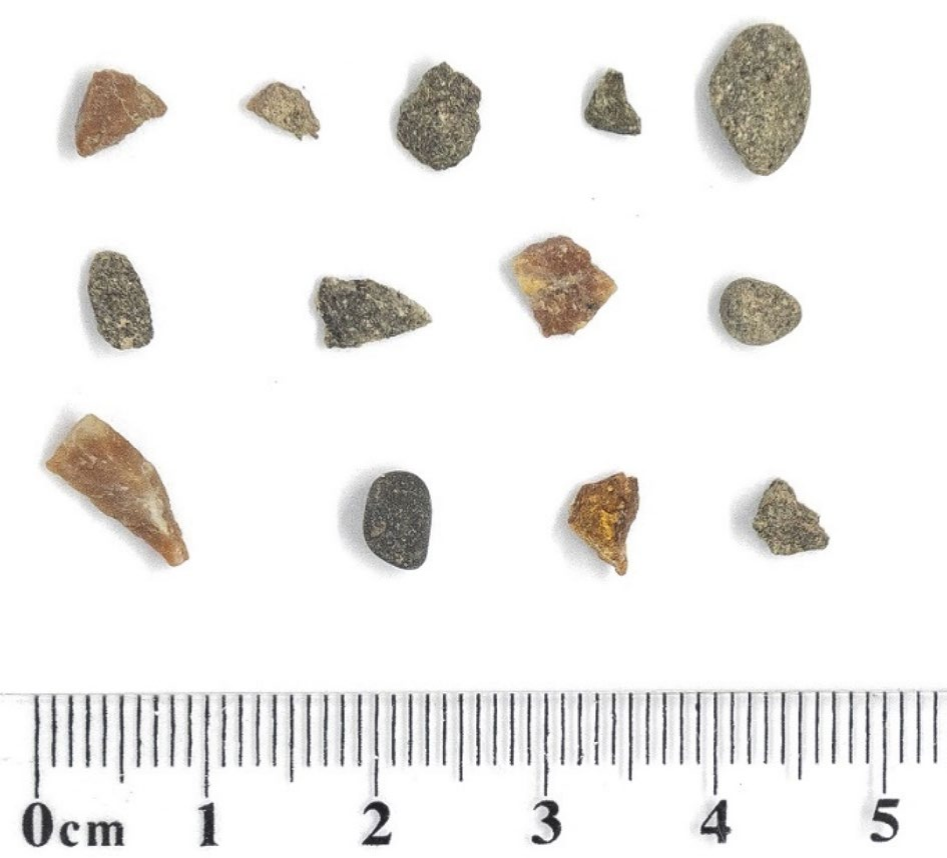
Photographs of pebbles Bruce manipulated and preened with which could be retrieved by the experimenters. Tools were retrieved by the experimenters only after Bruce dropped them.

Bruce’s pebble manipulations typically began with rolling a small pebble (Fig. 1) around with his tongue. He then wedged the pebble in his lower bill, and either rolled it or ran it along his feathers, which were held between the pebble and his tongue (Supplementary Video). This latter motion appears analogous to other subjects’ clasping and grinding of feathers between their upper and lower bills (Supplementary Video), which helps remove ectoparasites lodged between their barbs^36^.

**Figure 2:**
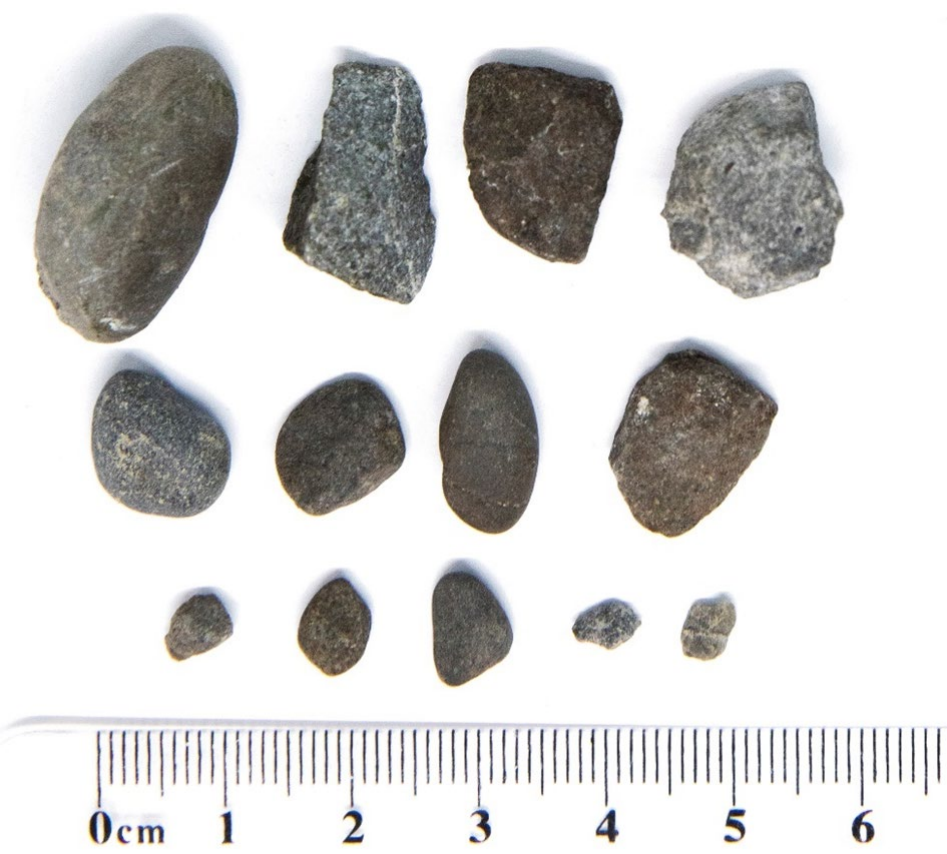
Photographs of pebbles and stones randomly sampled from the aviary by the experimenters.

We first examined whether or not Bruce’s pebble manipulations were followed by preening. Across 20 h of video observations recorded over 9 days, we recorded 30 videos where Bruce picked up a pebble and subsequently preened with it at least once (93.75% of cases), while there were only two instances where he picked up a pebble and did not preen with it for the duration of that observation. In one of these two instances, Bruce picked up a pebble and then took part in an aggressive display against another male, subsequently dropping it. In the other, he attempted to preen with the pebble but dropped it, then began interacting with another male. The frequency of his pebble interactions and preening behaviours provides strong evidence that Bruce’s preening behaviour was associated with his pebble manipulations; namely, when he interacted with a pebble, he was 1579.72 times more likely to preen with a pebble than not (Bayesian contingency table, n = 105, BF_10_ > 100, log_e_ odds ratio = 7.365).

### Are pebble tools valuable and effective to Bruce?

To determine whether Bruce’s pebble tooling was deliberate, we examined whether he attempted to retrieve or replace preening pebbles that he dropped during preening. We recorded 250 events where he dropped his preening pebble and subsequently retrieved or replaced it before he resumed preening, out of a total 262 retrieval and replacement events (95.42%). It is also notable that all pebbles used were similar in size (19.70 ± 9.14 mm^2^; Fig. 1), suggesting that his choice of pebble tools was deliberate and functional. To test this, we compared the pebble sizes Bruce used to a random sample of pebbles and stones in his environment (98.39 ± 75.62 mm^2^; Bayesian independent samples t-test, BF_10_ = 29.41; Fig. 2). This suggests that Bruce deliberately selected very small pebbles relative to the population of pebbles and stones present in the aviary.

In total, we observed 103 preening episodes by Bruce, with 30 involving a pebble and 73 not involving a pebble. This raises the question of whether Bruce preferred to preen with a pebble only in certain contexts. To this end, we compared the percentage of the time he spent preening different parts of his body (divided into wings, back, neck, chest, legs, and tail) with and without a pebble tool. Bruce employed pebbles for preening differentially throughout his body (Bayesian contingency table test, BF_10_ > 100; Supplementary Table 4). He preened most of his body parts without a pebble more frequently than with a pebble (wing: 29.72% vs. 9.94%; back: 8.39% vs. 5.00%; neck: 6.33% vs. 1.44%; chest: 11.22% vs. 3.33%; tail: 2.56% vs. 1.11%). However, he preened his legs without a pebble only 5.78% of the time, but with the pebble this constituted 15.17% of all observed preening behaviours suggesting that this body part was a specific focus of his pebble preening.

Finally, we compared the time Bruce spent preening with and without a pebble, to establish whether pebble use affected his preening time. Bruce preened himself for slightly longer with a pebble than without one (Bayesian independent samples t-test, BF_10_ = 1.26; with a pebble: 176.15 ± 208.51 s, without a pebble: 114. 28 ± 133.89 s).

### How does Bruce’s behaviour compare to that of other individuals?

**Figure 3:**
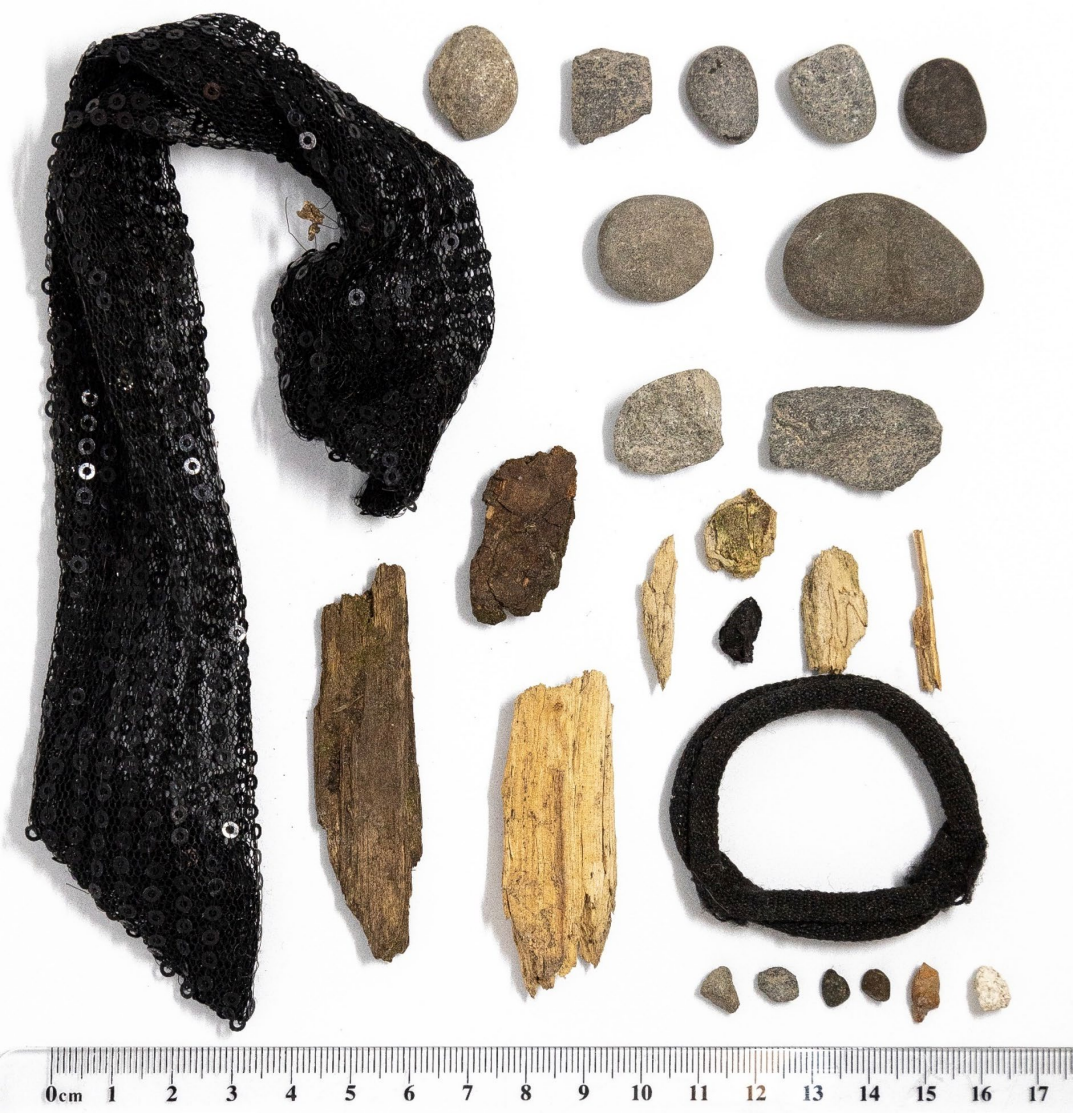
Photographs of objects that subjects other than Bruce interacted with and subsequently dropped, which experimenters were able to retrieve. There were no instances of these subjects preening whilst manipulating any objects.

**Figure 4:**
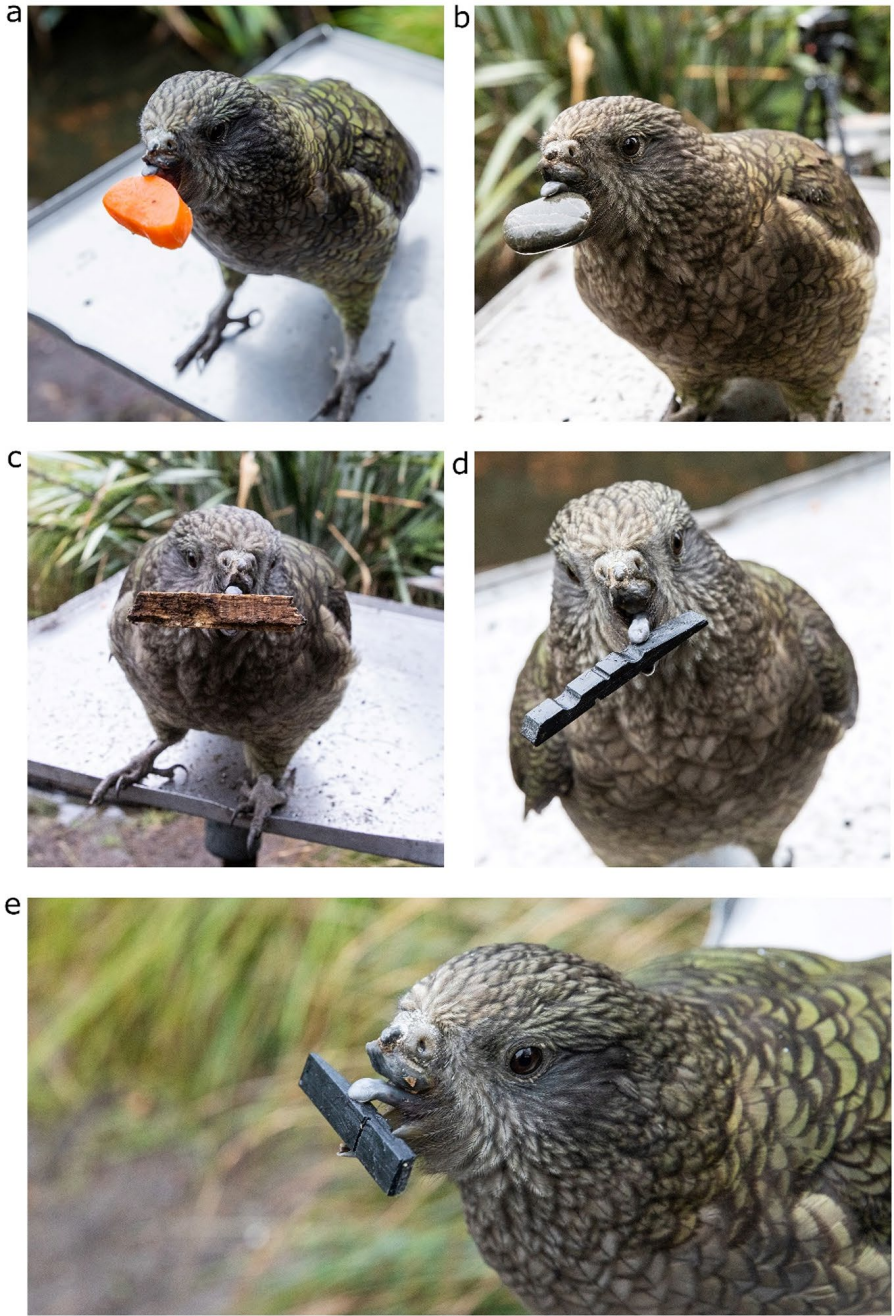
Photographs of Bruce handling objects larger than his preening pebbles, namely: (**a**) a slice of carrot, (**b**) a stone, (**c**) a piece of bark, (**d**) a black token used in previous cognitive experiments he was a part of. We also provide a close-up image in (**e**) demonstrating how he uses his tongue and lower mandible to hold these objects.

We compared Bruce’s manipulations of pebbles and non-pebble objects with those of other individuals. We observed no instances of any other subjects preening with pebbles or other objects, despite them regularly manipulating objects for purposes other than preening. Non-preening object manipulations occurred 202 times across eight other subjects (averaging 25.25 ± 37.53 instances each), compared to 18 times for Bruce. Therefore, Bruce appeared to manipulate non-pebble objects at a comparable or lower rate to other subjects (Supplementary Table 5) but was the only individual that used pebbles as preening tools. Interestingly, the size and types of objects that other subjects interacted with were also different, appearing to favor larger stones and non-pebble objects which were never used for preening (Bruce: 19.70 ± 9.14 mm^2^; other subjects: 184.92 ± 172.15 mm^2^; Bayesian independent samples t-test BF_10_ = 18.11; Fig. 3). This was despite the fact that Bruce can competently hold larger objects (Fig. 4).

## Discussion

We show that kea are capable of innovating self-care tooling, which is rarely seen in birds^28,29^. Our observations suggest that the preening pebble may be used as a flat surface against which Bruce can grind his feathers, such that the tongue and pebble act analogously to the upper and lower bills of other individuals. Bruce’s pebble preening behaviour strongly suggests deliberate tooling^31–35^. First, Bruce’s manipulations of pebbles were almost always followed by preening, suggesting that he picked up the pebble with the intent of using it as a preening tool. Second, Bruce often retrieved or replaced pebbles he lost or dropped, suggesting that he valued the pebble tool during preening. Third, Bruce selected pebbles of a particular size for use as tools, given that his pebble tools differed in size significantly from a randomly drawn sample of stones available in his environment. Fourth, while Bruce manipulated non-pebble objects at a similar rate to other individuals, he was the only subject ever observed preening with a pebble. Finally, when other did interact with stones and pebbles, they were significantly greater in size than those selected by Bruce. We therefore show that Bruce innovated pebble preening as a self-care tooling behaviour, likely as a direct consequence of his disability, by systematically testing the predictions for deliberate tooling in a bird species where tooling is not a species-specific behaviour.

Within the framework of tooling^1^, Bruce’s pebble preening can be described as an egocentric (self-directed) behaviour, with a single dynamic relation between the tool and his feathers or skin. The placing of the pebble is probably crucial to its functional use. Given that Bruce often repeatedly moves the pebble until he rests it on his lower mandible before he begins to preen with it, it is possible that its orientation is also important. However, this could also serve the purpose of lodging it comfortably within the mandible, unrelatedly to the function of the pebble. It is unclear if the alignment of the pebble is important. The properties of Bruce’s pebble tooling may therefore be different to those observed in foraging stick tooling by both kea and other parrot species^19,21,26,37^, which is allocentric and alignment-crucial, and the egocentric self-care tooling observed in a range of captive parrot species^9,10,30^. Further research is needed to investigate this possibility.

Our results suggest that Bruce’s tooling is flexible and deliberate. Evolved stereotyped tooling is innate, involving fixed action patterns that cannot be adapted to novel situations^4,38^. It is highly unlikely that Bruce’s pebble use is an evolved stereotyped action, given that, to the best of our knowledge, it is unique to him as an individual, and therefore likely a flexible, context-dependent innovation. Furthermore, Bruce does not attempt to preen with objects of a similar size in his environment to the pebble tools or with larger stones, as might occur with a fixed action pattern, despite being able to competently hold larger objects either between his tongue and lower bill, or between the remnants of his upper bill and lower bill.

It is unclear if the pebble tool employed by Bruce improves his preening efficiency, or whether it is adopted for some other function, given that preening episodes with the pebble were longer than those without. It might be that the pebble tool improves preening efficacy but not efficiency, for example by providing increased success in dislodging parasites at the cost of greater time expenditure. Bruce appeared to be selective in terms of the properties of the pebbles he chose, given both that he discarded some pebbles before attempting preening with them, and our data showing that he did not randomly sample from the environment. Precisely what pebble properties Bruce based his decisions on will be a focus of future work.

Together with kea’s other tooling innovations reported in the wild^21^ and in captivity^26^, Bruce’s deliberate self-care tooling suggests that kea may excel at innovating context-appropriate tools. This provides additional evidence for kea’s highly flexible problem-solving abilities, as evidenced in previous experiments including both captive and wild populations^21,26,27,37–42^. The ability to flexibly combine information in a domain-general manner^43^, when combined with playfulness and neophilic exploration of the environment, may be an important driver of technical innovations in kea and other species^44,45^. These results therefore support recent claims that tool use, including tooling, arises not only from the evolution of specialized physical cognition^46–49^, but can be innovated, when ecologically necessary, by species with sufficiently domain-general cognition^21,47–49^.

## Materials and methods

### Subjects and procedures

All observations were of a captive population of kea (*Nestor notabilis*) housed in a large, naturalistic outdoor aviary at Willowbank Wildlife Reserve in New Zealand. This population comprised thirteen individuals (10 males, 3 females) with ages ranging from 8 months to 25 years (Supplementary Table 1). Food and water were available ad libitum, and subjects were free to behave and interact normally in their environment throughout observation sessions. Observation sessions were at least one hour long and took place in the mornings, when birds were most active. This research was conducted under ethics approval from The University of Auckland Ethics Committee (reference number 001816). The study was also carried out in accordance with the relevant guidelines and regulations.

Observations took place in two stages. The first stage involved 20 h of focal observations of Bruce over nine days, where any interactions with objects and any preening episodes were recorded on video, until 30 s after the end of the preening or object manipulation episode. In every preening episode, experimenters either approached Bruce or zoomed in with their video camcorders to establish if an object was being held in his beak during the preening episode. This was unlikely to affect his behaviour, given that Bruce was familiar with all four experimenters and has an extensive history of participating in cognitive studies where he stands in close proximity to humans^40,43,50^. Bruce’s preening episodes were never interrupted, so that we could compare the total length of preening episodes with and without pebbles to assess whether pebble use affected preening efficiency.

The second stage involved 20 h of observations of the remaining 12 subjects in the aviary, over thirteen days. The experimenters watched the group and, whenever an individual began to preen or interact with objects, these episodes were recorded on video. At the end of each preening episode, or after two minutes of preening (whichever occurred first), the subject was interrupted and offered a black token, which they could exchange for a piece of food. This was done to inspect the subject’s mouth for any objects. We did not record entire preening episodes for other subjects, as we did not plan to compare the length of their preening episodes to Bruce’s. Most importantly, the observation of the other subjects allowed the experimenter to verify whether the subject had been holding any objects in their beak as they preened. Where the subject was perched out of reach, the video was zoomed into its beak until the experimenter could determine whether the subject was holding an object in its beak.

Wherever possible, across both observation stages, experimenters attempted to retrieve any objects subjects interacted with, either for preening or during other object manipulation behaviours. Experimenters also collected thirteen randomly selected pebbles and stones from across the aviary which were smaller than or equal to the largest stone manipulated by kea during our observation sessions. To ensure that pebbles were selected randomly, we drew a map of the aviary divided into 1 m^2^ squares with a grid. Thirteen coordinate locations were randomly generated, and the experimenter visited each of those in turn. Upon arrival in the middle of the pre-determined square, they closed their eyes and spun around. After opening their eyes, they took the pebble or stone closest to their right foot, provided it was smaller than or equal to the largest stone other subjects had interacted with in the past (Fig. 3). If it was bigger than the largest stone any of the kea had interacted with during observation sessions, the procedure was repeated until an appropriately sized stone was sampled. This ensured that we did not select any disproportionately large stones or rocks, which not even subjects with complete beaks would have been able to hold. Their surface areas were then measured digitally from photographs to compare Bruce’s pebble selections to a random subset of those available in the aviary.

### Video coding and analyses

Video data was coded using BORIS v.7.9.15 [Behavioral Observation Research Interactive Software]^51^. Behaviours were classed using a purpose-designed ethogram (Supplementary Table 2). Preening episodes were defined as lengths of time where the subject repeatedly touched their beak to any part of their body and ended when there was no beak contact for > 30 s. All observations were coded by one experimenter, while a second experimenter independently coded a random 10% sample of all recorded observations. Inter-coder agreement between experimenters was high for both stages, both in terms of preening behaviours and object manipulations (Supplementary Table 3).

To assess whether Bruce’s pebble manipulations co-occurred with preening, we used a Bayesian contingency table test with a Poisson sample distribution to compare his object manipulation frequency when object manipulation occurred alone or concurrently with preening. Second, we investigated whether the thirteen pebble tools selected by Bruce and recovered by the experimenters were deliberately selected for their properties by comparing their sizes to those of thirteen randomly selected pebbles from across the aviary, with a Bayesian independent samples t-test. We also assessed the frequency and duration of Bruce’s preening of different body parts with and without a pebble across a subset of observations where we could identify which body part was being preened. The frequency data were analyzed by a Bayesian contingency table test with a Poisson sample distribution. The duration was analyzed by a Bayesian paired samples t-test. Finally, we directly compared the objects manipulated by Bruce to those manipulated by other individuals using a Bayesian independent samples t-test. All main analyses were carried out in JASP v.0.14.1.0^52^ using default priors and all t-tests were two-sided.

## Supplementary Information

Supplementary Information.

Supplementary Video 1.

## Data Availability

Original data created for the study will be available on the following online repository upon publication: https://osf.io/j2sdn/.
