# Supplementary material for: Self-care tooling innovation in a disabled kea (Nestor notabilis)
Source: Sci Rep. 2021 Sep 10;11:18035. doi: 10.1038/s41598-021-97086-w (PMC8433200; doi:10.1038/s41598-021-97086-w)
Supplement: Supplementary file 1 — Supplementary Information. [file 41598_2021_97086_MOESM1_ESM.docx]

# Supplementary Materials for “Self-care tooling innovation in a disabled kea (*Nestor notabilis*)”

Amalia P. M. Bastos^1^, Kata Horváth^2,3,4^, Jonathan L. Webb^1^, Patrick M. Wood^1^, Alex H. Taylor^1^

^1^ School of Psychology, The University of Auckland, Private Bag 92019, Auckland 1142, New Zealand

^2^ Doctoral School of Psychology, ELTE Eötvös Loránd University, Izabella Utca 46, H–1064, Budapest, Hungary

^3^ Institute of Psychology, ELTE Eötvös Loránd University, Izabella Utca 46, H–1064, Budapest, Hungary

^4^ Brain, Memory and Language Research Group, Institute of Cognitive Neuroscience and Psychology, Research Centre for Natural Sciences, Magyar tudósok körútja 2, H–1117, Budapest, Hungary

## Subject Information

### Supplementary Table 1. Subject Information

| **Subject** | **Hatch Date**  **(Known or Estimated)** | **Sex** |
| --- | --- | --- |
| Angelina | October 2006 | F |
| Blofeld | August 2013 | M |
| Boh | September 2012 | F |
| Harley Quinn | August 2014 | F |
| Johnny | Unknown | M |
| Bruce | September 2012 | M |
| Loki | August 2014 | M |
| Megatron | October 2019 | M |
| Moriarty | August 2014 | M |
| Neo | September 2012 | M |
| Plankton | August 2014 | M |
| Spike | October 1995 | M |
| Taz | September 2012 | M |

Hatch dates and sex of individuals observed as a part of this study. All subjects were parent reared.

## Ethogram

### Supplementary Table 2. Behaviour Ethogram

| **Behaviour** | **Description** |
| --- | --- |
| Pebble Manipulation | Start and end times for all interactions with pebbles (defined as stones < 1cm diameter). |
| Non-pebble Object Manipulation | Start and end times for all interactions with objects other than pebbles. |
| Preening | Start and end times for instances of preening, that is, instances where a subject’s beak was in contact with any part of its body. Further labelled to distinguish preening in one of five body parts: wing, back, neck, chest, or tail. |

Videos were coded with three different state events, for which coders identified start and end times. The raw time stamp data was then used to calculate the duration of preening episodes with and without pebbles, as well as counts for instances of non-preening pebble manipulations, non-pebble object manipulations, and Bruce’s retrieval or replacement of preening pebbles within preening episodes.

## Inter-coder Reliability

Inter-coder reliability was analysed using the irr package [1] within R version 3.6.0 [2]. To assess the reliability of observations made by two experimenters on the random 10% subset of shared videos, we calculated a two-way random, absolute, average-measures intraclass correlation coefficient [*ICC*; 3]. Under this analysis, the degree of agreement between experimenters is evaluated using strict absolute differences and results can be generalised to any other potential observers possessing similar characteristics [4].

*ICCs* were generated for two pooled behavioural categories to maximise the sample size available: preening behaviour (comprising preening any of five body parts: wing, back, neck, chest, or tail) or object manipulation (comprising pebbles and non-pebble objects). Outputs were interpreted using previously cited thresholds [5], where *ICC* ﻿≥ 0.75 suggests excellent inter-coder agreement, ﻿0.6 ≤ *ICC* < 0.75 good agreement, ﻿0.4 ≤ *ICC* < 0.6 moderate agreement, and ﻿*ICC* < 0.4 poor agreement.

### Supplementary Table 3. Intraclass Correlation Coefficients

| **Observation Stage** | **Behaviour category** | **ICC** | **95% Confidence Intervals** |
| --- | --- | --- | --- |
| 1 and 2 | Preening | 0.894 | 0.767 - 0.952 |
| 1* | Pebble and Non-pebble Object Manipulations | 0.925 | 0.790 - 0.974 |

**ICCs* could not be generated for object manipulation in stage 2 due to limited variance.

Intraclass correlation coefficients for coder’s identification of preening behaviours (all body parts, with and without pebbles) and object manipulation behaviours (including pebbles and non-pebble objects).

## Supplementary Results

|  | | | **Pebble Use** | | | |  | |  |
| --- | --- | --- | --- | --- | --- | --- | --- | --- | --- |
| **Modifiers** | |  | **No** | | **Yes** | | **Total** | |  |
| Back |  | Count |  | 151 |  | 90 |  | 241 |  |
|  |  | % of total |  | 8.39 % |  | 5.00 % |  | 13.39 % |  |
| Chest |  | Count |  | 202 |  | 60 |  | 262 |  |
|  |  | % of total |  | 11.22 % |  | 3.33 % |  | 14.56 % |  |
| Leg |  | Count |  | 104 |  | 273 |  | 377 |  |
|  |  | % of total |  | 5.78 % |  | 15.17 % |  | 20.94 % |  |
| Neck |  | Count |  | 114 |  | 26 |  | 140 |  |
|  |  | % of total |  | 6.33 % |  | 1.44 % |  | 7.78 % |  |
| Tail |  | Count |  | 46 |  | 20 |  | 66 |  |
|  |  | % of total |  | 2.56 % |  | 1.11 % |  | 3.67 % |  |
| Wing |  | Count |  | 535 |  | 179 |  | 714 |  |
|  |  | % of total |  | 29.72 % |  | 9.94 % |  | 39.67 % |  |
| Total |  | Count |  | 1152 |  | 648 |  | 1800 |  |
|  |  | % of total |  | 64.00 % |  | 36.00 % |  | 100.00 % |  |
| BF_10_Poisson  N | | | | |  | 1.321e+58  1800 | | |  |
|  | | | | | | | | |  |

### Supplementary Table 4. Counts for Bruce’s Preening of Different Body Parts with and without a Pebble Tool

Bayesian contingency table including counts (total n = 1800) of all of Bruce’s preening bouts as categorised by body parts (back, chest, leg, neck, wing) and use of a pebble tool (yes or no). We found strong evidence (BF_10_ > 100) for differences in the distribution of preening behaviour across the five categories.

| **Individual** | **Number of Object Manipulations Observed** | **BF_10_** |
| --- | --- | --- |
| Boh | 1 | 0.667 |
| Harley | 1 | 0.667 |
| Loki | **125** | **1.661e +32** |
| Megatron | **24** | **7647.734** |
| Moriarty | **8** | **2.069** |
| Neo | **39** | **6.385e +7** |
| Plankton | 3 | 0.533 |
| Taz | 1 | 0.667 |
| Angelina | 0 | - |
| Blofeld | 0 | - |
| Johnny | 0 | - |
| Spike | 0 | - |
| Bruce | 2* |  |

Supplementary Table 5. Comparisons of non-pebble object manipulation frequencies between Bruce (Stage 1) and other individuals (Stage 2)

To examine whether the frequency of Bruce’s object manipulations was comparable to those of other birds living in the aviary, we conducted separate Bayesian binomial tests between Bruce and each individual. Out of the 12 other birds, four were not seen to manipulate objects, four manipulated objects at a similar frequency to Bruce (BF_10_ < 1), one showed a slightly higher number of object manipulations (1 < BF_10_ < 3), and three manipulated objects more frequently than Bruce (BF_10_ > 100). *To standardise the observation duration for all individuals across Stages 1 and 2, as only Bruce was observed for Stage 1 whereas 12 birds were observed for Stage 2, we divided the total number of Bruce’s object manipulations (18) by 12 (rounded to a whole number). This was performed under the assumption that object manipulations were equally likely to occur across in Stages 1 and 2, and that each individual in Stage 2 was observed for an equal amount of time.

**Supplementary Video**

The supplementary video contains: (a) real-time footage of typical preening behaviour by a kea, (b) slow motion footage of Bruce using a pebble to preen his chest, (c) real-time footage of Bruce using a pebble to preen his legs, and (d) real-time footage of Bruce selecting a preening pebble.

## Supplementary References

[1] Gamer, M., Lemon, J. & Singh, P. IFP. Irr: Various Coefficients of Interrater Reliability and Agreement. (2012).

[2] Team RC. R: A Language and Environment for Statistical Computing. (2019).

[3] Hallgren, K. A. Computing inter-rater reliability for observational data: an overview and tutorial. *Tutor. Quant. Methods Psychol.* **8**, 23 (2012).

[4] Koo, T. K. & Li, M. Y. A guideline of selecting and reporting intraclass correlation coefficients for reliability research. *J. Chiropr. Med.* **15**, 155–163 (2016).

[5] Cicchetti, D. V. Guidelines, criteria, and rules of thumb for evaluating normed and standardized assessment instruments in psychology. *Psychol. Assess.* **6**, 284 (1994).
